# Molecular Mechanisms Contributing to the Impairment of Steroid Hormones, Sperm Characteristics, and Testicular Architecture in Male Rabbits After Chronic Exposure to Cadmium: Role of Gallic Acid and Selenium as Antioxidants

**DOI:** 10.3390/toxics13040323

**Published:** 2025-04-21

**Authors:** Salah A. Sheweita, Saleh M. Al-Qahtani, Rofida M. Ahmed, Mohamed S. Sheweita, Ahmed Atta

**Affiliations:** 1 Institute of Graduate Studies and Research, Alexandria University, Alexandria 21526, Alexandria Governorate, Egypt; rofida.mohamed555@yahoo.com; 2 Department of Clinical Biochemistry, College of Medicine, King Khalid University, Abha 62521, Aseer Province, Saudi Arabia; 3 Department of Child Health, College of Medicine, King Khalid University, Abha 62521, Aseer Province, Saudi Arabia; smuadi@kku.edu.sa; 4 Southmead Hospital, Southmead Road, Westbury-on-Trym, Bristol BS10 5NB, UK; mohammedsheweita@gmail.com; 5 Faculty of Agriculture, Alexandria University, Alexandria 21545, Alexandria Governorate, Egypt; ahmed75atta@yahoo.com

**Keywords:** cytochrome P450, antioxidant enzymes, free radicals, infertility, cadmium

## Abstract

One hazardous material that occurs naturally in the environment and induces oxidative stress is cadmium (Cd). Epidemiological data revealed that exposure to cadmium in the workplace and environment might be linked to many illnesses and serious testicular injuries. Aims: It is taught that antioxidants can protect different organs against environmental toxic compounds. Therefore, the current investigation aims to show the role of antioxidants (gallic acid and selenium) in the protection against cadmium toxicity, including the architecture of the testes, semen properties, steroid hormones, protein expression of cytochrome P450 [CYP 19 and 11A1] contributing to the production of steroid hormones, and antioxidant enzyme activities, in male rabbits. Methods: Male rabbits were given cadmium orally three times/week [1 mg/kg BW] for twelve weeks. In addition, gallic acid (20 mg/kg) or selenium (1 mg/kg BW) was administered two hours before cadmium treatment. This investigation included a spectrophotometer, histopathology, and Western immunoblotting techniques. Results: Cadmium treatment significantly reduced sperm counts, testosterone, and estrogen levels after four, eight, and twelve weeks of treatment. In addition, after a 12-week treatment of rabbits with cadmium, the activity of 17β-hydroxysteroid dehydrogenase and antioxidant enzymes, including catalase, superoxide dismutase, glutathione reductase, glutathione peroxidase, and glutathione S-transferase, as well as the glutathione levels, were inhibited in the testes tissue. On the other hand, following cadmium treatment, rabbit’s testes showed a discernible increase in free radical levels. Interestingly, the activity of antioxidant enzymes and level of free radicals were recovered in rabbits treated with gallic acid or selenium before cadmium treatment. In addition, after 12 weeks of cadmium treatment, the steroidogenic protein expressions of CYP 11A1 and CYP 19 were upregulated and downregulated in the testes, respectively. Interestingly, after pretreatment of rabbits with either gallic acid or selenium for two hours before cadmium administration, the downregulated CYP11A1 was restored to normal levels. In the histopathological investigation, immature spermatozoids and sloughed spermatogonium cells were observed in cadmium-treated rabbits’ testes. On the other hand, pretreatments of rabbits with gallic acid or selenium mitigated and alleviated the adverse effects of cadmium on testes architecture and increased the production of healthy sperm. Conclusions: The lower levels of steroid hormones could be due to the downregulation of CYP11A1, inhibition of 17β-hydroxysteroid dehydrogenase, antioxidant enzyme activities, and the induction of free radical levels. Furthermore, the pretreatment of rabbits with gallic acid or selenium mitigated the adverse effects of cadmium on the tissue architecture of testes and steroid hormone levels.

## 1. Introduction

Many diseases, including infertility, can be brought on by environmental pollutants [1,2]. Cadmium is one of the most environmentally harmful metals that causes a serious threat to the reproductive system [3,4,5] since there is a strong correlation between cadmium exposure and male infertility [6,7,8,9]. In addition, another study demonstrated that sixty infertile adult males (20 with azoospermia and 40 with oligospermia) had higher cadmium levels in blood and semen than the 40 controls with normal sperm counts [10]. Also, higher cadmium levels were found in the blood of 501 infertile couples in Rockville, USA, which suggests that cadmium levels adversely affected human fertility [11]. In addition, high cadmium levels in testicular blood systems in males with varicocele were found to increase sperm cell death in the testes [12]. As per a recent study, the offspring of zebrafish exhibited reproductive damage because their parents were exposed to cadmium [13].

In adult female crayfish, selenium reversed the suppression of ovarian development caused by cadmium and improved ovary index and oocyte development [14]. Selenium inhibited cadmium-induced programmed necrosis of chicken testicular Leydig cells [15]. The combination of selenium with Bisphenol A (an endocrine disruptor) increased antioxidant enzyme activity, increased the activity of 3β- and 17β-hydroxysteroid dehydrogenases, and improved testicular and epididymal sperm characteristics in rats [16]. In addition, selenium therapy decreased acrylamide-induced testicular oxidative stress, inflammation, and apoptosis in rats while increasing the protein expression of reproductive enzymes, sperm motility, and morphology [17]. The effect of selenium nanoparticles (SeNPs) coated with gallic acid (GA) on azoospermic rats induced by busulfan showed a substantial increase in testosterone levels, antioxidant status, testicular tissue characteristics, and sperm parameters [18]. In addition, gallic acid protects the mouse ovary against doxorubicin-induced oxidative damage by enhancing glutathione (GSH) concentrations, increasing mitochondrial activity, and reducing inflammation and apoptosis through modulation of the PI3K and mTOR signaling pathways [19]. Furthermore, gallic acid protects against aflatoxin B1-mediated histological lesions in epididymis, hypothalamus, sperm characteristics, and hormone levels associated with reproductive function in rats [20].

Low male fertility is mainly due to low sperm count, aberrant sperm morphology, and low motility [21]. Nevertheless, 15% of men experienced infertility with normal sperm characteristics. Therefore, other sperm parameters are required to identify male infertility [22]. One of these parameters is oxidative stress, which induces apoptosis and DNA fragmentation in sperm [23,24]. Several factors, such as lifestyle choices (drinking and smoking), some malignancies, pathological and genetic factors, and environmental toxins, are known to induce oxidative stress and consequently induce DNA fragmentation in the sperm [25]. Induction of oxidative stress decreased the rate of embryonic development, implantation, pregnancy, and in vitro and in vivo fertilization [26]. It has been demonstrated that oxidative stress occurs due to the production of free radicals in the seminal fluid in large quantities, disrupting sperm function [26,27]. Therefore, the elimination of free radicals from the seminal fluid by nutritional antioxidants and induction of antioxidant enzyme activities, including superoxide dismutase, glutathione S-transferase, glutathione peroxidase, and glutathione reductase, has been found to improve human fertility [28].

Moreover, establishing and maintaining male sexual function depends on testosterone levels since low testosterone levels cause male sexual dysfunction and infertility [29,30]. The main source of testosterone in males is the Leydig cell [31]. Steroid hormone synthesis is primarily mediated by cytochrome P450 isozymes, specifically CYP11A, CYP11B1, and CYP11B2 proteins bound to the mitochondrial membrane and CYP17, CYP19, and CYP21 proteins bound to the endoplasmic reticulum (microsomal) membrane [32,33,34]. The inner mitochondrial membrane contains the enzyme cytochrome P450 side-chain cleavage (P450 SCC), where the steroidogenic acute regulatory protein (StAR) transports cholesterol from the outer mitochondrial membrane [35,36,37]. P450 SCC produces pregnenolone from cholesterol, which is then converted to testosterone in the smooth endoplasmic reticulum by CYP17, CYP21, 3-hydroxysteroid dehydrogenase (3-HSD), and 17β-hydroxysteroid dehydrogenase (17β-HSD) [36]. The 17β-HSD enzyme, required to synthesize testosterone, is almost exclusively expressed in the human testes [38]. Therefore, male pseudohermaphroditism has been associated with the inhibition of 17 β-HSD activity as well as the downregulation of the protein expression of cytochrome P450 isozymes [39,40].

As far as we know, no previous study has investigated the mechanisms underlying the decrease in steroid hormone levels after cadmium therapy [CYP 19 and 11A1], which are involved in the steroidogenesis of steroid hormones and are studied following cadmium treatment alone or two hours of pretreatment with selenium or gallic acid before cadmium treatment. In addition, oxidative stress markers, activity of antioxidant enzymes, semen characteristics, and testicular architecture were assayed in rabbits.

## 2. Materials

ABCAM Pharmaceuticals, United Kingdom, provided the Western blotting detection kit, primary anti-rabbit antibodies for CYP 19 and CYP 11A1 (Cat. Nos. PA1-21398 and MO-AB-07770Y, respectively), and secondary antibody-antirabbit-HRP (Cat. No. ab6721). The Sigma Chemical Company, Saint Louis, MO, USA, provided the remaining chemicals.

### 2.1. Animals

The Poultry Research Centre, Breeding Rabbit Section, Faculty of Agriculture, Alexandria University, Egypt, provided thirty male New Zealand white rabbits that were seven months old and weighed 2.5–3.5 kg. The local committee on animal care at Alexandria University in Egypt (AU082303261132) approved the experimental design and techniques, which adhere to the guidelines of the National Institutes of Health. The rabbits were housed in an animal facility with good ventilation, a temperature of 22 °C, a relative humidity of 40–60%, a 12-h light/dark cycle, and free access to a pellet-based diet. Before beginning the experiment, the rabbits were acclimatized for one week and separated into six equal groups.

### 2.2. Research Design

Groups I and II were administered 0.5 mL of saline and cadmium chloride, respectively, via oral gavage three times a week (2 mg/kg body weight). In groups III and IV, rabbits received gallic acid (20 mg/kg) and sodium selenite (1 mg/kg). Before cadmium administration for two hours, groups V and VI received gallic acid and sodium selenite three times/week. Cadmium, selenium, and gallic acid doses were determined according to the previous studies [41,42].

## 3. Methods

### 3.1. Preparation of the Microsomal Fraction

Rabbits were anesthetized by intramuscular xylazine injections (10 mg/kg) after the twelfth week of different treatments [43]. The testes were taken out and dried after being rinsed in a cold 0.1 M potassium phosphate buffer (pH 7.3). The testes were homogenized in three volumes of 0.1 M phosphate buffer (pH 7.3) at 4 °C [44]. The testicular homogenates were centrifuged for 20 min at 4 °C and 12,000× *g* to extract nuclei and cell debris. For biochemical examination, 1.5 mL of supernatant was kept at −80 °C. The remaining supernatant was ultracentrifuged at 105,000× *g* for 60 min at 4 °C to separate the microsomal pellets. Finally, microsomal pellets containing cytochrome P450 isozymes were suspended in 0.1 M potassium phosphate buffer (pH 7.3) and frozen at −80 °C [44].

### 3.2. Biochemical Assays

Lowry et al.’s (1951) method has been used to measure total protein content [45]. Bogovich and Payne’s (1980) techniques were used to determine the specific activity of 17β-hydroxysteroid dehydrogenase (EC 1.1.1.51) [46]. In brief, 100 μL of 0.5 mM NAD, 100 μL of S9 fraction as an enzyme source, and 2.7 mL of 50 mM Tris-HCl buffer pH 9.0 were added to 100 μL of 1.0 mM testosterone as a substrate. The reaction mixture was kept at 37 °C for ten min. To terminate the reaction, 100 μL of 0.1 M HCl was added. The control tubes, with the same reagents as the sample tubes but no sample added, received 100 μL of 0.1 M HCl before the S9 fractions were added. NADH absorption was measured at 340 nm. Enzyme activity was measured as μmol of NADH/minute/mg protein. The amount of reduced glutathione in the homogenate of testes tissue was determined using bis-(3-carboxy-4-nitrophenyl)-disulfide for color development and sulfosalicylic acid for protein precipitation [47]. The glutathione reductase activity was assessed by measuring NADPH oxidation at 340 nm. One nanomole of NADPH oxidized/min/mg protein represents one unit of enzyme activity [47].

Glutathione S-transferase [GST] activity was evaluated using the method of Lee et al. (1981) [48]. The conjugate of GSH with l-Chloro-2, 4-Dinitrobenzene (CDNB) was measured at 340 nm with a double-beam spectrophotometer. A unit of enzyme activity refers to the quantity of enzyme required to catalyze the synthesis of 1 mmol of CDNB conjugate/mg protein/min under laboratory conditions. GST activity is calculated using the molar extinction coefficient of 9.6 mM^−1^ cm^−1^. The activity of the glutathione peroxidase enzyme (GPx; EC. 1.11.1.9) was measured using the procedure described by Chiu et al. in 1976 [49]. The catalase activity (CAT; EC1.11.1.6) in the supernatant of testes homogenates was measured using Luck’s 1974 method [50]. The molar absorbance coefficient was used to determine the quantity of H_2_O_2_ breakdown over a certain duration at a wavelength of 240 nm. Catalase activity is expressed as a unit per mg of protein.

Misra and Fridovich’s (1972) methods were employed to determine the activity of superoxide dismutase (SOD; EC 1.15.1.11) in the testis homogenate-supernatant [51]. Xanthine and xanthine oxidase produce superoxide radicals, which react with nitro tetrazolium blue (NTB) to yield formazan dye, which is used to assess SOD activity. The concentration of formazan dye was measured using a spectrophotometer at 560 nm. This enzyme’s inhibition level was determined in micromoles per minute per milligram protein. Malondialdehyde (MDA) in testes, the end product of lipid peroxidation, was measured as a thiobarbituric acid reactive substance (TBARS) using Tappel and Zalkin’s 1959 method [52]. At 532 nm, the color intensity of MDA was assessed. The concentrations of TBARS are calculated using an extinction coefficient of 156,000 M^−1^ Cm^−1^.

### 3.3. Semen Analysis

Over the 12 weeks of the experiment, ejaculations were obtained using an artificial vagina and placed in a clean, wide glass container. The sample was incubated at 37 °C for semen liquefaction. Within 30 to 60 min of semen collection, liquefaction was followed by semen analysis to prevent dehydration or temperature changes that could impair the quality of the semen.

### 3.4. Sperm Motility

Sperm motility test results were estimated according to the method of Atashfaraz et al., 2013 [53]. At 27 °C, 10 µL of the sperm suspension was put on a sterile, warmed slide and then covered with a coverslip. After that, slides were examined at a power of a hundred times their original size with a Leica DM 750 light microscope on a stage that had been preheated to 37 °C.

### 3.5. Sperm Count

Freund and Carol’s (1964) method has been used to count the number of sperms [54]. After collecting 50 µL of ejaculate, it was diluted with 2 mL of normal saline and warmed to 37 °C. The spermatozoa were then suspended and diluted with a normal saline solution. Using a Pasteur pipette, 200 µL of the suspension was pipetted into each of the two Neubauer hemacytometer chambers, allowing capillary action to fill them. The spermatozoa were then counted under a light microscope (Leica DM 750).

### 3.6. Sperm Morphology

To investigate sperm morphology, a drop of sperm solution was placed on a glass slide. Following drying, the suspension was stained with 1% eosin. A light microscope was used to evaluate spermatozoa anomalies in the head, middle, and tail of each rabbit at a magnification of 400× [55].

### 3.7. Blood Samples

Throughout the experiment, about 3 mL of blood was collected from each rabbit’s marginal ear vein once every four weeks. The blood was subsequently mixed with a clot activator to separate the serum. The clear serum was obtained after 5 min of centrifugation at 3000× *g* and stored at −80 °C for further research. The activities of serum Aspartate Aminotransferase (AST) (U/L) and serum Alanine Aminotransferase (ALT) (U/L) were determined [56]. Urea and creatinine levels were determined using Patton and Crouch’s 1977 technique [56].

### 3.8. Hormonal Assays

The testosterone and estrogen levels were measured using solid-phase enzyme immunoassay (ELISA) kits (Diagnostics Biochem, Canada Inc., London, ON, Canada). To stimulate an immune response, 20 μL of serum was placed in wells containing magnetic bead-immobilized antibodies. After removing the excess mixture, the substrate and fluorescent reagent mixtures were incubated at 37 °C. The fluorescence intensity at 940 nm was measured using a spectrophotofluorometer [53].

### 3.9. Western Immunoblotting for the Identification of the Immobilized Proteins

The sample application buffer was mixed with fifty µg of protein samples, pooled from each group, and heated for three minutes before being applied to the gel. Upon completion of the electrophoretic process, the gel was removed. Proteins were transferred from the gel onto the nitrocellulose membrane. After the protein bands were transferred, the membranes were removed and washed twice with TBS for 15 min. Goat-raised primary antibodies against rabbit CYP19 and CYP11A1 were added to the membrane at a 1:1000 dilution in TBS buffer. The membranes were incubated with a secondary antibody (anti-goat IgG-HRP) at a dilution of 1:500 in TBS buffer. The mixture of peroxide buffer and luminal/enhancer was applied to the membrane’s surface as an immunodetection solution. An X-ray film was used to detect the fluorescent protein bands after removing excess substrate [57].

### 3.10. Histopathology

Small testicular tissues from each rabbit were collected and stored in a 10% buffered formalin solution. After being sectioned at 4–6 µm and fixed in paraffin, the sections were treated with conventional grades of xylol and alcohol. Slides containing sections from different groups were stained with hematoxylin and eosin (H&E) and examined under a light microscope (Leica DM 750) [58].

### 3.11. Statistical Analyses

The statistical program SPSS 16 was used to determine means, standard deviations, and standard errors. Using a one-way ANOVA, the significance values between groups were set at *p* < 0.05 and/or *p* < 0.001.

## 4. Results

### 4.1. Cadmium and Steroid Hormones

In the present investigation, serum testosterone levels decreased after 4, 8, and 12 weeks of cadmium treatment (Table 1). However, these lowered testosterone levels were increased when selenium or gallic acid was administered to rabbits before cadmium administration but did not reach normal levels (Table 1). In addition, the motility and viability of the sperm and the estrogen levels were decreased in the cadmium-treated group compared to the control group (Table 1). It is interesting to note that pretreating rabbits with selenium or gallic acid at 4 weeks reversed the decreased estrogen levels caused by cadmium, but at 8 and 12 weeks, estrogen levels increased and did not return to normal (Table 1). Additionally, following 4, 8, and 12 weeks of cadmium treatment, the semen quantities (ejaculates), motility, and counts decreased (Table 1). However, rabbits pretreated with gallic acid or selenium before cadmium treatment showed increased ejaculate, motility, and sperm counts, but these were not restored to normal values (Table 1).

### 4.2. Effect of Cadmium on 17β-Hydroxysteroid Dehydrogenase Activity and the Protein Expression of Cytochrome P450 Isozymes in Testes Tissues

**Figure 1:**
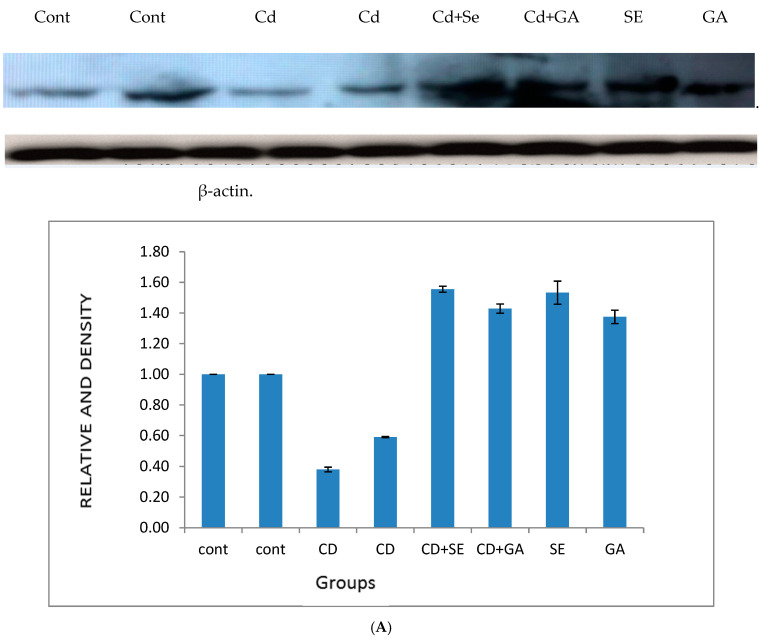

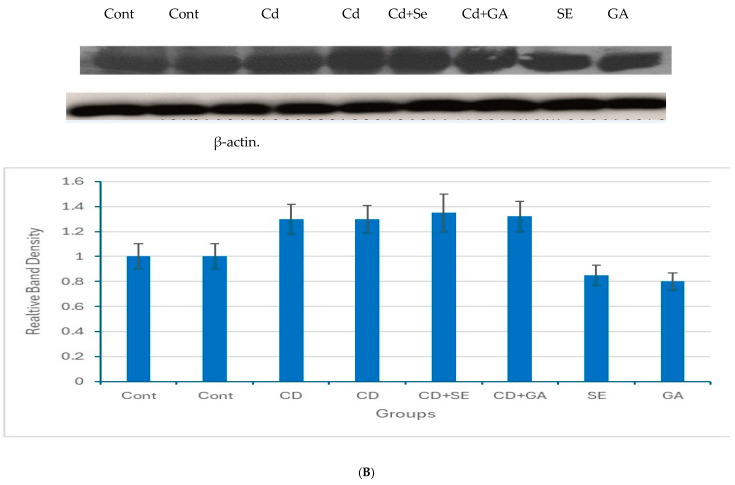
(**A**) Western immunoblotting data showed the changes in the protein expression of CYP11A1 in the testes of male rabbits after treatment with cadmium, cadmium + selenium, cadmium + gallic acid, selenium, and gallic acid-treated groups, respectively. The band density of each treatment was calculated using the Quantity One program. (**B**), Western immunoblotting data showed the changes in the protein expression of CYP19 in the testes of male rabbits after treatment with cadmium, cadmium + gallic acid, cadmium + selenium, and gallic acid and selenium-treated groups, respectively. The band density of each treatment was calculated using the Quantity One program.

In the present study, cadmium treatment decreased the activity of 17β-hydroxysteroid dehydrogenase in testes after 12 weeks (Table 2). However, compared to the control group, selenium treatment in rabbits surpasses gallic acid in restoring the decreased activity of this enzyme caused by cadmium (Table 2). Furthermore, the protein expressions of CYP 19 and CYP 11A1 were upregulated and downregulated, respectively, after 12 weeks of cadmium treatment in testes tissues (Figure 1A,B). Interestingly, pretreatment of rabbits with selenium or gallic acid for two hours before the administration of cadmium recovered the downregulated CYP11A1 protein expression to normal levels (Figure 1A). However, such treatments could not recover the upregulated CYP 19 protein expression to normal levels (Figure 1B).

### 4.3. Cadmium and Antioxidant Enzyme Activities in Testes Tissues

The free radical levels increased in the testes as determined by thiobarbituric acid reactive compounds after cadmium treatment (Table 2). However, pre-treated rabbits with gallic acid or selenium before cadmium administration restored the elevated levels of free radicals to their normal level (Table 2). Additionally, the testes tissues of the cadmium-treated group showed decreased activity of antioxidant enzymes, including reduced glutathione (GSH), glutathione peroxidases (GPx), catalase (CAT), glutathione S-transferase (GST), glutathione reductase (GR), and superoxide dismutase (SOD) (Table 2). Remarkably, selenium pretreatment before cadmium administration mitigated the inhibitory effects of cadmium on SOD, GST activity, and GSH levels and restored their activities to normal levels (Table 2). Although the other antioxidant enzyme activities were elevated in the rabbits pretreated with gallic acid before cadmium administration compared to the cadmium-treated group, they did not reach their normal levels (Table 2). The lowered activity of antioxidant enzymes (SOD, GST, and GPx) caused by cadmium was restored to normal levels much more efficiently by selenium than by gallic acid (Table 2).

In serum, the concentrations of urea and creatinine and the activity of alanine transaminase (ALT) and aspartate transaminase (AST) were significantly increased in the cadmium-treated group compared to the control group (Table 2). However, pretreatment of rabbits with selenium before cadmium administration restored the creatinine level and ALT activity to their normal levels. In contrast, urea concentration and AST activity could not be recovered (Table 2). On the other hand, pretreatment of rabbits with gallic acid before cadmium treatment decreased urea, creatinine, AST, and ALT but could not restore them to their normal levels (Table 2).

### 4.4. Histopathology of Testicular Tissues

**Figure 2:**
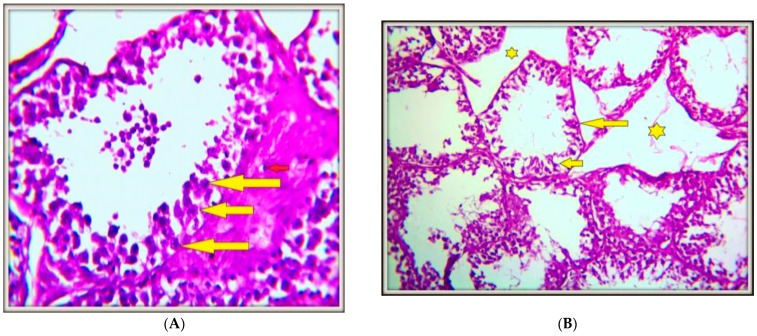

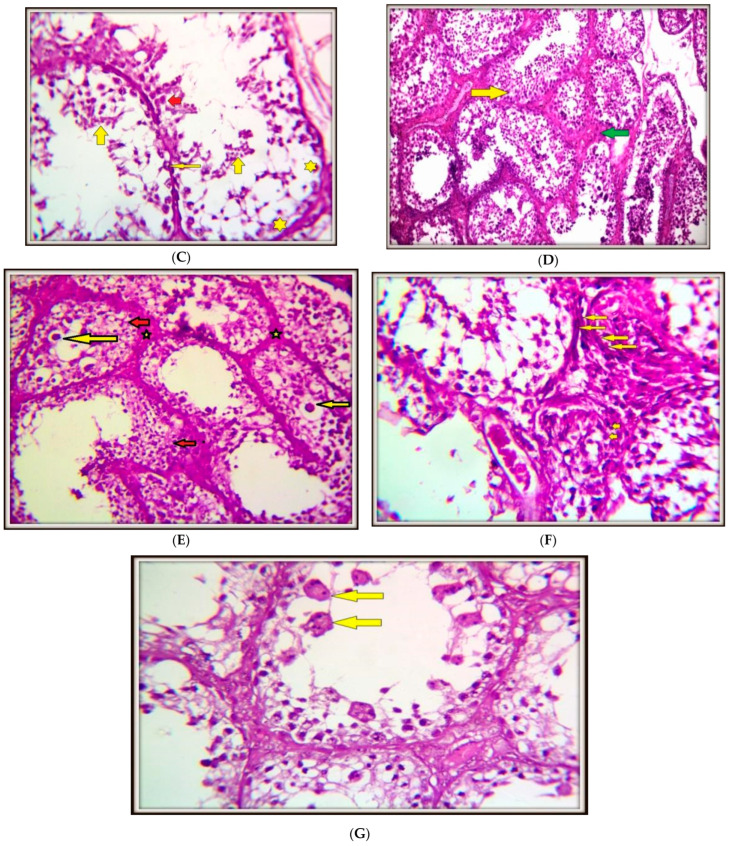
(**A**) An examination of the testes from male rabbits in the control group using histopathology revealed normal, intact testis tubules and undamaged interstitial tissues. The Leydig cells found in the interstitial tissues have normal vacuolated lipid droplets (short red arrow). They have big euchromatin stippling chromatin-differentiated nuclei (yellow arrows) (SainH&EX400); (**B**) Examining the testes of male rabbits who received cadmium treatment demonstrated a significant loss of spermatogenic cells and necrosis of all cells, as indicated by small, dark nuclei and shrinking cells (short arrow). The thinned basement membrane of the testis tubule (long arrow), significant damage of interstitial tissues (stars), and complete loss of germinal epithelial layers in some tubules (StainH&EX200); (**C**) Higher magnification of the cadmium-treated rabbits showed severe loss of spermatogenic cells and germinal epithelial layer cells in testis tubules. The basement membrane showed complete sloughing of spermatogonium cells (stars), and the remaining cells showed severe necrobiotic changes that were represented by small cells with shrinking nuclei (thin yellow arrow) and other cells with complete loss of their nuclei (red arrows), with sloughed necrotic cells (thick yellow arrow) in the center (StainH&EX400); (**D**) Histopathological study of testes of male rabbits treated with gallic acid showed highly intact testes, highly dividing spermatogonium cells (yellow arrow), and intact interstitial tissues (green arrow) (StainH&EX200); (**E**) Histopathological study of testes of male rabbits treated with selenium showed proliferation of myoid cells (spindle-shaped peritubular cells) and proliferation of Leydig cells (long yellow arrows and short thick yellow arrows) (H&EX200), red arrow and star(small cells with shrinked nuclei); (**F**) Rabbit testis pretreated with gallic acid before administration of cadmium (Cd+G) showed an improvement in growing germinal epithelial layers and intact interstitial tissues with the presence of some multinucleated giant cells (long yellow arrows), which indicated the accumulation of degenerated spermatogenic cells (StainH&EX400), short yellow arrow (degenerative changes to the interstitial compartment and seminiferous epithelium); (**G**) Rabbit testis pretreated with selenium before administration of cadmium (Cd+Se) showed tubules with regenerated spermatogenic cells and the presence of many multinucleated giant cells (yellow arrows), which contain degenerated spermatogonium cells but are still vacuolated (stain H&Ex400).

Histological analysis of testis tissues was carried out to confirm the modifications in the above biochemical results. The typical testicular architecture of the control group is seen in Figure 2A following their administration of saline solution. Following cadmium delivery, spermatogenic cells with significant vacuolization and the presence of damaged immature spermatozoa were found (Figure 2B). Also, in the cadmium-treated group, the basement membrane displayed complete spermatogonium cell (star) sloughing, but the remaining cells displayed small cells with shrinking nuclei (thin yellow arrow), and other cells displayed complete nuclei loss (red arrows) with necrotic cells (thick yellow arrow) (Figure 2C). Rabbits treated with gallic acid only exhibited a large number of spermatogenic stem cells residing on the basement membrane, along with normal chromatin and normal mitotic division in their testes (Figure 2D). In the testis tissues of selenium-treated rabbits, the number of sperm increased, and normal meiosis and mitotic divisions were also shown (Figure 2E). Rabbits pretreated with gallic acid before administration of cadmium (Cd + G) showed an improvement in growing germinal epithelial layers and intact interstitial tissues with the presence of some multinucleated giant cells (long yellow arrows), which indicated accumulation of degenerated spermatogenic cells in testis tissues (Figure 2F). The testes of rabbits pretreated with selenium before administration of cadmium (Cd + Se) showed tubules with regenerated spermatogenic cells and the presence of many multinucleated giant cells (yellow arrows) that contain degenerated spermatogonium cells but are still vacuolated (Figure 2G).

## 5. Discussion

Testosterone is generally recognized for its critical function in maintaining reproductive organ maturation, spermatogenesis, the establishment of secondary sexual characteristics, and sexual behavior regulation [59]. A population study found a negative connection between blood Cd levels and serum testosterone concentrations [59]. However, the probable molecular mechanism by which ambient cadmium suppresses testicular testosterone production is unknown. The Interaction of several toxic compounds with the hormone receptors inhibited enzyme activities involved in the steroidogenesis of steroid hormones [44,60,61]. The present study showed that chronic cadmium treatment for 4, 8, and 12 weeks decreased testosterone and estrogen levels, semen volumes, and sperm counts. Remarkably, these changes were restored when selenium or gallic acid was given to rabbits before cadmium administration. Supporting our findings, it has been confirmed that Cd suppressed testicular testosterone levels in mice [59]. Furthermore, the female rats treated with cadmium had low estrogen levels in their serum, whereas the groups treated with cadmium plus vitamin C and cadmium plus vitamin E showed higher estrogen levels [62]. Moreover, gallic acid alone increased testosterone levels in rats [63]. In the present study, sperm motility decreased in cadmium-treated rabbits. The decrease of sperm motility might be due to the inhibition of tyrosine phosphorylation of 55–57 KDa proteins, which function as an engine to impede intracellular energy metabolism [64]. In addition, cadmium induces higher abnormality in sperm heads than lead [65]. Supporting our findings, it has been found that sperm parameters, testicular tissue properties, and testosterone levels significantly improved when selenium nanoparticles coated with gallic acid (GA) were administered together before the administration of busulfan, which decreased these parameters [18].

The synthesis of steroid hormones in the testis of rabbits is significantly influenced by the expression of the CYP11A1 protein and 17-hydroxysteroid dehydrogenase activity [36]. Therefore, the mechanism underlying the decrease in testosterone levels in rabbits treated with cadmium might be due to suppression of 17-hydroxysteroid dehydrogenase activity and downregulation of CYP11A1 protein expression. In agreement with the present study, in the hens treated with cadmium, the gene expression of the CYP450 was reduced in their livers [66]. The current investigation showed that pretreatment of rabbits with either gallic acid or selenium restored the cadmium-induced down-regulation of CYP11A1 protein expression. Also, in pig livers, selenium administration markedly raised the expression and activity of CYP1A2 and CYP2D25 and lowered CYP3A29 [67]. Furthermore, selenium increases the expression of CYP19A1 in caprine ovarian granulosa cells [68].

Androgens can be turned into estrogen hormones by the enzyme CYP19. Therefore, CYP19 is an essential enzyme for estrogen synthesis [69]. In the present study, pretreatment of rabbits with either gallic acid or selenium before cadmium administration upregulated the protein expression of CYP19, which might explain the high level of estrogen hormone in these groups compared to the cadmium-treated rabbits. This induction might be due to the interaction of gallic acid with aromatic hydrocarbon receptors responsible for the induction of cytochrome P450 content [70]. Supporting this suggestion, molecular docking and in vitro studies showed that gallic acid (GA) acts as a novel aromatic hydrocarbon receptor ligand that significantly activates cytochrome P450 [70].

Many antioxidants in the seminal fluid play an important role in sperm protection from reactive oxygen species (ROS). It has been shown that high ROS levels could reduce fertility by triggering sperm death, DNA damage, and lipid oxidation [71,72,73]. Spermatozoa are vulnerable to the harmful effects of ROS due to their low antioxidant content levels [74]. Furthermore, high ROS levels induce oxidative stress, which is primarily present in tissues with low antioxidant enzyme activity, including SOD, CAT, GR, GPx, and GST [71,72]. In the current investigation, cadmium therapy decreased glutathione levels and inhibited SOD, GSH, GST, CAT, GSR, and GPx activities. These inhibitions increased free radical levels and oxidative stress in the rabbit testes of cadmium-treated rabbits. Selenium is a cofactor for GPx and SOD, and its interaction with cadmium may be a possible mechanism of their inhibition [75]. Supporting this finding, administration of selenium to rabbits before cadmium boosts the activities of SOD and GPx [30]. This effect may be due to the availability of selenium in a significant amount, which is an important element for the GPx and SOD to carry out their maximum activity. Therefore, antioxidants may be a useful strategy for reducing the burden of male infertility caused by oxidative stress [73,76]. Consistent with our findings, the inhibition of antioxidant enzyme activity was restored when rats were pretreated with curcumin and Nigella sativa oil before cadmium treatment [77,78].

Depletion of glutathione levels and inhibition of antioxidant enzyme activity may also be an explanation for low testosterone and sperm count in rabbits treated with cadmium. In agreement with the present study, selenium showed protective effects in reducing cadmium-induced damages, including mitochondrial damage, oxidative stress, and apoptosis by raising the antioxidant capacity in L8824 cells [30]. Also, in lead-treated testes of rats, it was found that another antioxidant, diallyl sulfide, enhanced the decline in epididymal sperm count and motility, spermatogenesis score, and serum testosterone levels [79]. Moreover, selenium, gallic acid, and zinc have recently been found to strengthen the antioxidant defense against cadmium toxicity in Helianthus annuus seedlings via attenuation of inflammation and oxidative stress pathways [80,81].

Cadmium-treated rabbit testes were examined histopathologically, which revealed vacuolization and the presence of disturbed immature spermatozoa. In the cadmium-, gallic acid-, and selenium-treated groups, healthy sperm, spermatogenic, and normal mitotic and meiotic divisions were obtained. Furthermore, pretreatment of rabbits with selenium or gallic acid reversed the effects of cadmium treatment on the quantity and motility of sperm in the testes compared to the control group. Cadmium treatment has been noted to exhibit decreased sperm counts and increased sperm aberrant forms, which is consistent with the current investigation [82,83,84,85,86].

It is interesting to note that antioxidant supplements follow the concept of Hormesis, by which small, non-toxic stresses or mild stress can be used to induce cellular adaptive responses that protect biological systems against subsequently large and potentially lethal stresses of the same, similar, or different nature. Therefore, gallic acid and selenium can be considered hormetic nutrients as they can induce healthy effects by activating antioxidant pathways (i.e., SOD, GST, GSH, GPx) to prevent or attenuate oxidative damage caused by cadmium. Consistent with this observation, emerging evidence shows that low doses of hormetic drugs and/or functional food nutrients can upregulate the antioxidant Nrf2 pathway and redox resilience proteins (i.e., GSH, SOD, and CAT) to inhibit or block oxidative damage [87,88,89]. On the other hand, a high dose of drugs or antioxidant compounds can be toxic to cells and tissues, leading to increased oxidative stress markers and subsequent inhibition of antioxidant proteins, resulting in the onset and progression of disorders associated with oxidative stress [90,91,92]. The field of hormetic/adaptive responses activated by natural supplements in enhancing endogenous redox defense systems is emerging as a promising preventive and therapeutic strategy in several disorders, including infertility caused by exposure to environmental pollutants [87,88,89,90,91,92].

Conclusion: Cadmium treatment was found to reduce testosterone levels, downregulate CYP21A isozyme protein expression and 17-hydroxysteroid dehydrogenase activity involved in steroidogenesis, inhibit antioxidant enzyme activities, and enhance free radical levels. In rabbits pretreated with gallic acid and selenium before cadmium administration, the adverse effects of cadmium on these parameters were alleviated. Therefore, individuals exposed to cadmium through various industrial occupations may use gallic acid or selenium supplements.

## Figures and Tables

**Table 1:** Changes in levels of testosterone, estrogen, and semen parameters at 4, 8, and 12 weeks after pretreatment of male rabbits with selenium and gallic acid, before administration of cadmium for two hours.

Treatments						
Weeks	Control	Cadmium	Cadmium + Gallic Acid	Cadmium + Selenium	Gallic Acid	Selenium
**Testosterone level (ng/dL)**						
**4**	5.72 ± 0.11 ^a^	5.0 ± 0.10 ^b^	5.8 ± 0.06 ^a^	6.04 ± 0.09 ^a^	6.12 ± 0.06 ^a^	6.08 ± 0.23 ^a^
**8**	6.26 ± 0.01 ^a^	4.20 ± 0.08 ^d^	5.04 ± 0.08 ^c^	5.34 ± 0.07 ^b^	6.28 ± 0.06 ^a^	6.38 ± 0.06 ^a^
**12**	6.46 ± 0.09 ^b^	3.38 ± 0.05 ^d^	4.92 ± 0.12 ^c^	4.76 ± 0.05 ^c^	6.54 ± 0.06 ^b^	6.88 ± 0.09 ^a^
**Estrogen (ng/dL)**						
**4**	10.26 ± 0.12 ^b^	8.30 ± 0.14 ^d^	9.76 ± 0.16 ^c^	10.120 ± 0.09 ^bc^	10.76 ± 0.12 ^a^	10.96 ± 0.16 ^a^
**8**	10.48 ± 0.10 ^b^	5.40 ± 0.14 ^e^	8.84 ± 0.24 ^d^	9.34 ± 0.12 ^c^	11.40 ± 0.07 ^a^	11.32 ± 0.11 ^a^
**12**	11.26 ± 0.10 ^b^	4.12 ± 0.07 ^d^	8.88 ± 0.28 ^c^	8.54 ± 0.09 ^c^	11.70 ± 0.05 ^a^	11.80 ± 0.12 ^a^
	**The volume of Siemens (Ejaculate) (mL)**					
**4**	0.640 ± 0.024 ^a^	0.460 ± 024 ^c^	0.600 ± 0.031 ^ab^	0.520 ± 0.020 ^bc^	0.620 ± 0.037 ^a^	0.600 ± 0.031 ^ab^
**8**	0.670 ± 0.023 ^a^	0.54 ± 0.024 ^c^	0.62 ± 0.21 ^ab^	0.54 ± 0.23 ^c^	0.66 ± 0.024 ^a^	0.58 ± 0.020 ^bc^
**12**	0.70 ± 0.03 ^ab^	0.64 ± 0.024 ^ab^	0.66 ± 0.024 ^ab^	0.62 ± 0.02 ^b^	0.70 ± 0.031 ^ab^	0.72 ± 0.02 ^a^
**pH of Siemens**						
**4**	7.5 ± 0.031 ^b^	7.7 ± 0.037 ^a^	7.7 ± 0.031 ^a^	7.5 ± 0.02 ^a^	7.6 ± 0.020 ^b^	7.5 ± 0.02 ^b^
**8**	7.5 ± 0.20 ^b^	7.7 ± 024 ^a^	7.7 ± 0.02 ^a^	7.7 ± 0.02 ^a^	7.6 ± 0.031 ^b^	7.5 ± 0.024 ^b^
**12**	7.5 ± 0.02 ^b^	7.7 ± 0.037 ^a^	7.6 ± 0.037 ^a^	7.6 ± 0.024 ^a^	7.5 ± 0.024 ^b^	7.5 ± 0.024 ^b^
**Sperm concentration (10^6^/mL)**						
**4**	168 ± 1.77 ^b^	123 ± 1.7 ^d^	135 ± 1.5 ^c^	133 ± 1.6 ^c^	168 ± 1.6 ^b^	189 ± 1.7 ^a^
**8**	186 ± 2.3 ^a^	111 ± 3.1 ^d^	134 ± 1.8 ^c^	148 ± 2.4 ^b^	185 ± 3.4 ^a^	192 ± 2.6 ^a^
**12**	190 ± 2.5 ^a^	100 ± 3.6 ^c^	141 ± 2.5 ^b^	146 ± 2.9 ^b^	193 ± 2.9 ^a^	198 ± 3.8 ^a^
**Motility**						
**4**	70 ± 1.5 ^b^	38 ± 1.7 ^d^	62 ± 1.3 ^c^	59 ± 1.1 ^c^	75 ± 1.3 ^a^	72 ± 1.5 ^ab^
**8**	74 ± 1.7 ^a^	24 ± 2.04 ^c^	56 ± 1.3 ^b^	55 ± 1.7 ^b^	76 ± 1.5 ^a^	76 ± 1.6 ^a^
**12**	77 ± 1.7 ^a^	20 ± 1.5 ^c^	52 ± 1.4 ^b^	50 ± 1.9 ^b^	79 ± 1.6 ^a^	82 ± 1.3 ^a^
**Vitality%**						
**4**	85.58 ± 1.47 ^a^	72.44 ± 1.11 ^b^	84.44 ± 1.14 ^a^	72.74 ± 1.26 ^b^	83.92 ± 1.57 ^a^	87.20 ± 1.77 ^a^
**8**	80.76 ± 1.44 ^a^	44.74 ± 2.09 ^d^	74.06 ± 1.7 ^b^	66.42 ± 1.65 ^c^	78.10 ± 1.83 ^ab^	81.62 ± 1.99 ^a^
**12**	78.32 ± 1.94 ^ab^	26.44 ± 2.95 ^d^	75.20 ± 1.16 ^b^	61.68 ± 1.90 ^c^	76.10 ± 1.56 ^ab^	81.82 ± 1.81 ^a^

All values were presented as the mean and standard error of five rabbits for each treatment. Means with different superscript letters were statistically significant. Means with the same superscript letter were not statistically significant. The significance level for mean differences was set at *p* < 0.05.

**Table 2:** Changes in the activity of 17β-hydroxysteroid dehydrogenase and antioxidant enzymes in the testes of rabbits and liver and kidney markers in their serum after pretreatment with selenium and gallic acid for two hours before cadmium administration.

Enzymes	Treatments					
	Control	Cadmium	Cadmium + Gallic Acid	Cadmium + Selenium	Gallic Acid	Selenium
	In Testes Tissues					
17 β-hydroxysteroid dehydrogenase (Unit/mg protein/min)	2.59 ± 0.109 ^a^	0.76 ± 0.04 ^b^	1.8 ± 0.048 ^d^	2.1 ± 0.0244 ^e^	2.6 ± 0.048 ^a^	3.8 ± 0.037 ^c^
Superoxide dismutase activity (U/mg protein)	143.3 ± 6.09 ^b^	68.5 ± 6.28 ^d^	112.2 ± 1.97 ^c^	129.7 ± 4.60 ^b^	167.2 ± 3.65 ^a^	176.5 ± 1.97 ^a^
Glutathione S-transferase (GST) (U/mg protein)	2.88 ± 0.073 ^c^	1.66 ± 0.024 ^e^	2.56 ± 0.107 ^d^	2.88 ± 0.058 ^c^	4.60 ± 0.100 ^a^	3.98 ± 0.058 ^b^
Glutathione reductase (µmol/g tissue)	20.96 ± 1.88 ^c^	12.42 ± 0.73 ^e^	15.22 ± 1.18 ^de^	18.46 ± 1.50 ^cd^	26.78 ± 1.17 ^b^	35.54 ± 1.03 ^a^
Thiobarbituric acid reactive substances (µmol/g tissue)	2.60 ± 0.09 ^c^	3.97 ± 0.03 ^a^	2.74 ± 0.01 ^c^	2.57 ± 0.01 ^c^	1.48 ± 0.10 ^d^	0.75 ± 0.03 ^e^
Glutathione peroxidase (GPx) (U/mg protein)	70.70 ± 1.58 ^c^	41.12 ± 1.14 ^f^	65.44 ± 1.43 ^d^	71.56 ± 1.65 ^e^	93.22 ± 1.68 ^a^	84.80 ± 1.22 ^b^
Glutathione level (µmol GSH/g tissue)	5.98 ± 0.058 ^c^	3.82 ± 0.020 ^f^	5.91 ± 0.03 ^c^	5.90 ± 0.1 ^c^	7.90 ± 0.01 ^a^	6.94 ± 0.024 ^b^
Catalase activity (H_2_O_2_/mg protein/min)	64.6 ± 1.52 ^c^	29.7 ± 1.31 ^e^	41.8 ± 1.88 ^d^	47.6 ± 2.08 ^d^	67.2 ± 1.06 ^c^	94.4 ± 2.05 ^a^
In Serum						
Urea (mg/dL)	27.60 ± 1.72 ^d^	84 ± 3.19 ^a^	66.20 ± 2.55 ^b^	51 ± 1.14 ^c^	30.6 ± 2.31 ^d^	33.80 ± 1.85 ^d^
Creatinine (mg/dL)	0.86 ± 0.05 ^d^	1.8 ± 0.10 ^a^	1.16 ± 0.04 ^b^	0.94 ± 0.05 ^cd^	1.10 ± 0.03 ^bc^	1.06 ± 0.02 ^bc^
Alanine Transaminase [ALT] (unit/L)	78.40 ± 4.40 ^c^	147.80 ± 5.5 ^a^	103.20 ± 6.31 ^b^	82 ± 4.77 ^c^	70.20 ± 4.74 ^c^	72 ± 9.92 ^c^
Aspartate Transaminase [AST] (unit/L)	56.80 ± 3.67 ^d^	222.80 ± 4.4 ^a^	85.80 ± 4.52 ^c^	114 ± 4.51 ^b^	36 ± 3.24 ^e^	37 ± 3.0 ^e^

All values were presented as the mean and standard error of five rabbits. Means with different superscript letters were statistically significant. Means with the same superscript letter were not statistically significant. The significance level for mean differences was set at *p* < 0.05.

## Data Availability

The data that support the findings of this study are available from the corresponding author upon reasonable request.
